# Diurnal and Reproductive Stage-Dependent Variation of Parental Behaviour in Captive Zebra Finches

**DOI:** 10.1371/journal.pone.0167368

**Published:** 2016-12-14

**Authors:** Boglárka Morvai, Sabine Nanuru, Douwe Mul, Nina Kusche, Gregory Milne, Tamás Székely, Jan Komdeur, Ádám Miklósi, Ákos Pogány

**Affiliations:** 1 Department of Ethology, Eötvös Loránd University, Budapest, Hungary; 2 Behavioural and Physiological Ecology, Groningen Institute for Evolutionary Life Sciences, University of Groningen, Groningen, Netherlands; 3 Department of Biology and Biochemistry, University of Bath, Bath, United Kingdom; Rutgers The State University of New Jersey, UNITED STATES

## Abstract

Parental care plays a key role in ontogeny, life-history trade-offs, sexual selection and intra-familial conflict. Studies focusing on understanding causes and consequences of variation in parental effort need to quantify parental behaviour accurately. The applied methods are, however, diverse even for a given species and type of parental effort, and rarely validated for accuracy. Here we focus on variability of parental behaviour from a methodological perspective to investigate the effect of different samplings on various estimates of parental effort. We used nest box cameras in a captive breeding population of zebra finches, *Taeniopygia guttata*, a widely used model system of sexual selection, intra-familial dynamics and parental care. We investigated diurnal and reproductive stage-dependent variation in parental effort (including incubation, brooding, nest attendance and number of feedings) based on 12h and 3h continuous video-recordings taken at various reproductive stages. We then investigated whether shorter (1h) sampling periods provided comparable estimates of overall parental effort and division of labour to those of longer (3h) sampling periods. Our study confirmed female-biased division of labour during incubation, and showed that the difference between female and male effort diminishes with advancing reproductive stage. We found individually consistent parental behaviours within given days of incubation and nestling provisioning. Furthermore, parental behaviour was consistent over the different stages of incubation, however, only female brooding was consistent over nestling provisioning. Parental effort during incubation did not predict parental effort during nestling provisioning. Our analyses revealed that 1h sampling may be influenced heavily by stochastic and diurnal variation. We suggest using a single longer sampling period (3h) may provide a consistent and accurate estimate for overall parental effort during incubation in zebra finches. Due to the large within-individual variation, we suggest repeated longer sampling over the reproductive stage may be necessary for accurate estimates of parental effort post-hatching.

## Introduction

Parental care, in its widest sense (including pre-natal care such as for instance searching for a safe place to lay eggs in insects), is an essential component of individual reproduction in most animal species. It can be provided by either or both parents in various forms and at different reproductive stages [1, 2]. The recognition of parental care’s central role in development, life-history, sexual selection and intra-familial conflict has placed this trait in the focus of evolutionary biology [3–5]. It is essential for empirical studies of parental care to quantify relevant behavioural traits accurately. However, to date surprisingly little research has been directed at investigating the combined effects of within-individual and within-pair variation and the applied sampling method on the accuracy and reliability of estimates of parental effort from a methodological perspective.

In birds (where biparental care predominates [6]), there is a great diversity in the most frequently applied sampling methods used to quantify parental care [7]. Previous studies frequently monitored time spent in the nest and number of nest visits during incubation and/or nestling provisioning (e.g. [8, 9–12]). In the majority of these studies the nest is either directly observed, or more frequently, video-recorded for behavioural coding from the inside or outside for a pre-defined period at a standardized time (i.e. starting at a given time of a given day of the reproductive stage). The majority of methods implemented in these studies seems arbitrarily chosen (i.e. without providing any scientific reason for why the given sampling method was applied) and are not validated for accuracy or reliability [13, 14]. Although standardization may decrease the potential effects of within-individual variation on comparing overall parental effort, without a detailed analysis several important questions remain unaddressed. For instance, do we need multiple sampling bouts from a reproductive stage? How long should the observation period be? When should the observation period start? Monitoring parental behaviour can be seen as an optimization problem because it is costly both in terms of time and research effort, whereas statistical power increases asymptotically with sample size. We should therefore aim at a trade-off that maximizes the return for observer effort [14]. Another question that standardization does not address is whether a sample obtained by observing care at an arbitrarily chosen time and day of the reproductive stage is representative for other periods of the day or other days of the reproductive stage. For instance, in Kentish plovers, *Charadrius alexandrinus*, the total parental effort (male + female), its variability and the division of care between the sexes have all been shown to change over the course of the day in response to variation in the need to cool the eggs with daily temperature fluctuations [15]. Parental behaviour may also change both with reproductive value of offspring (expected to increase with clutch or brood age) and the needs of offspring (expected to decrease with clutch or brood age) [1, 3]. Therefore, an arbitrarily chosen time and duration of period may not yield valid conclusions regarding division of parental sex roles and total parental effort from many aspects.

In this study, we investigated patterns of parental behaviour from a methodological perspective in standard laboratory conditions using a captive population of zebra finches, *Taeniopygia guttata*. This species has emerged as a widely used model system of sexual selection, intrafamilial conflicts and parental strategies [7, 16–19]. Despite its importance, we have very little information with regard to within-pair variation of parental effort (but see Gilby et al. [7] for a detailed analysis of variation in feeding visits and allocation of food). Moreover, the methods used to quantify behaviour are diverse (e.g. [7, 10, 20–26]) and there is no consensus as to which method provides the most accurate estimates, limiting inference among studies. Here we quantify diurnal and reproductive stage-dependent variation in parental behaviour between and within individuals based on video-recordings of nest box cameras taken at different stages of incubation and nestling provisioning. 12h (recorded during early and late in incubation and nestling provisioning) and 3h (recorded in the middle of the reproductive stages) continuous recordings were taken. These recordings were then broken up for different sampling windows to test how sampling at different times of the day and different days of the reproductive stage influence predictions of overall daily parental effort and division of labour between the sexes. Our main questions were: (1) does a 1h sampling window at a random time of day provide equally accurate prediction of daily parental effort compared to a 3h sampling window, based on the extent of within-individual diurnal and reproductive stage-dependent variation? We chose 3h and 1h sampling windows based on a random selection of ten publications with non-automated sampling of parental care during incubation and nestling provisioning in zebra finches [10, 20, 22, 24, 26–31]. These studies used 3.2 ± 1.8 h (mean ± SD, range: 0.5–8 h) sampling windows, and we intended to compare the average sampling window with one from the lower range. (2) Does parental effort during early incubation and nestling provisioning predict parental effort during late incubation and nestling provisioning, respectively? (3) Does parental effort during incubation predict parental effort during offspring provisioning? We answered these questions focusing on some of the most widely investigated forms of parental effort, including time allocated to incubation, brooding and nest attendance by each sex separately and together, and the number of feeding visits.

## Materials and Methods

### Study Population and Housing Conditions

We used 20 adult males and 20 adult females from the zebra finch population kept at the Animal House of Eötvös Loránd University, Hungary. This population was established from the domesticated stock maintained at Bielefeld University (Germany) [32]. Birds were randomly selected for this study from sexually mature birds (mean ± SD age: 1.6 ± 0.9 years; range: 134 days– 3.3 years; zebra finches are fully mature at around 100 days post-hatching [18]). Most of them were already experienced breeders (mean ± SD n of breedings: 0.9 ± 1.4; range: 0–5). Subjects wore one numbered aluminium ring (Principle Kft., Újlengyel, Hungary) for individual identification. Males and females were randomly paired and housed in cages of 100 x 30 x 35 cm, with a wooden nest box (12 x 12 x 12 cm) attached to the outside of the cage. Coconut fibres were provided for nest material. A 14:10 h light:dark cycle (lights on at 6:00, local time) was maintained using full-spectrum tube lights (NASLI, Prague, Czech Republic) connected to a timer. Temperature was kept constant at 20–21°C using air conditioning.

Birds were provided with *ad libitum* access to food and water. Seed mixture consisted of equal portions of three different types of millet (*Panicum miliaceum luteum*, *P*. *miliaceum rubrum*, *P*. *italicum*) and canary grass (*Phalaris canariensis*), and a small portion (less than 1%) of Niger seed (*Guizotia abyssinica*). In addition, a daily provision of egg-food (Egg food tropical finches, Orlux, Versele-Laga, Belgium) and germinated seeds (home-made from the above seed mixture) were provided for protein and vitamin supply.

### Recording Parental Behaviour

We collected data during early, middle and late incubation of 10 pairs and during early, middle and late nestling provisioning of another set of 10 pairs (different sets of nests were used for the two reproductive stages due to experimental considerations of another study). Nests that were recorded during nestling provisioning were also recorded for 3h (between 10:00–13:00, see below) during middle incubation to analyse between-reproductive stage variation in parental effort. Nest boxes were monitored every second day to establish start of egg laying, start of incubation and hatching. In most cases, this allowed the incubation or hatching date to be established, but in instances where the date was uncertain, embryo development (determined by lamping eggs with a small led light) or nestling size was necessary to ascertain the date. Zebra finches, similar to many small passerines, lay one egg per day during egg-laying. Two to three days of hatching asynchrony is common in captive zebra finches because females usually begin incubation after laying two-three eggs. We considered the reproductive stage as post-hatching from the date when the first egg hatched in a given clutch.

**Fig 1:**
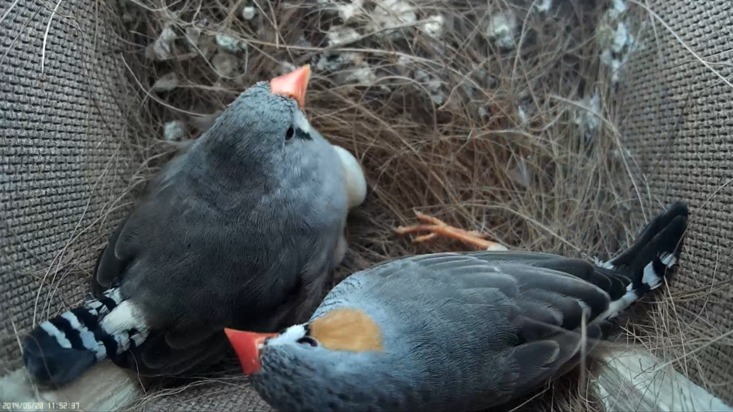
Parental behaviour of breeding zebra finches were recorded using small Mobius digital cameras. The wide field of view coupled with the ability to record videos in low light conditions and a pronounced sexual dimorphism in this species ensured that parental sex and activity inside the nest could be determined accurately.

Parental behaviour was recorded inside the nest boxes using four small digital cameras (Mobius Action Cam, JooVuu Store, UK) with wide-angle lenses (116° field of view). The wide-angle lens covered almost the entire inside of the nest box, allowing close monitoring of parental behaviour. The camera was configured to record a coloured, full HD video in low-light conditions, and this resulted in good quality recordings from which parental sex and behaviour could be established unambiguously (Fig 1).

A same-size dummy camera, made of wood and painted black to resemble the Mobius camera, was mounted on top of the nest box lids at all times except for filming to habituate the birds to the device. The night before recording, the dummy camera was replaced with the real camera, with its objective inserted into a hole in the lid. The camera was connected to a 5V adapter and a timer, and was programmed to record when powered externally. The use of dummy cameras and the timer ensured that recording did not interfere with the birds’ parental behaviour so that we can use the whole sample (cf. [13]). To avoid potential data loss, the camera was set to save 4 GB video segments (maximum setting for video clip length) in.mov format on a 64 GB microSD card.

In our population, hatching of nestlings starts from day 13–14 of incubation, followed by approximately 20 days of nestling provisioning before fledging. Nests were therefore recorded on day 3, day 8 and day 13 of incubation (calculated as days spent from the first day of incubation) or day 3, day 10 and day 17 post-hatching (calculated as days spent from hatching of the eldest i.e. first-hatched nestling in the nest), representing early, middle and late reproductive stages. On days 3 and 13 of incubation and days 3 and 17 of nestling provisioning, nests were recorded for a full day between 07:00–19:00, whereas on day 8 of incubation and day 10 of nestling provisioning, nests were recorded for three hours between 10:00–13:00. The 10 nests that were recorded during nestling provisioning were also recorded on day 8 of incubation for three hours to test whether parental effort is repeatable between reproductive stages. Taking smaller samples (3h instead of 12h) in the middle of the reproductive stages significantly decreased time and effort of behavioural coding (resulting in a total of 570hs as opposed to 840hs of recordings, i.e. 32% less), while allowing information to be gathered for the middle of each reproductive stage. Due to a technical failure, we lost data for a ca. 1.5h interval during a full-day recording on day 13 of incubation at a nest, resulting in varying sample sizes among some analyses.

### Behavioural Coding and Dataset Compilation

Recordings were behaviourally coded using Solomon Coder (v 15.11.19, developed by András Péter [33]). This software works based on a one video–one coding sheet protocol, so the first step was to merge the corresponding 4 GB video segments recorded by the Mobius camera using QuickTime for Windows (v. 7.7.9, by Apple Inc.).

From the recordings, we coded the following behaviours for each sex separately: present inside the nest box, incubation, and brooding or feeding nestlings. We considered a bird to be inside whenever a part of its body was seen on the recording but was not incubating eggs or brooding the nestlings. Incubation or brooding refers to the bird either sitting on eggs or young, or being in body contact with them. When a bird sat beside its already-incubating or brooding pair, we considered that this bird too was incubating or brooding because its body heat likely contributed to warming the eggs or nestlings.

For each video recording, two coding sheets were produced in Solomon Coder and from these two Excel output sheets were generated. The first coding sheet and output contained markers set every hour, and the second contained markers set every three hours. This allowed us to calculate summarized data for all recordings using 1h or 3h sampling windows as periods. Therefore, a full-day recording consisted of 12 periods and 4 periods with 1h or 3h sampling windows, respectively. We also calculated overall daily parental effort for the full length of recordings. Four datasheets, containing information on parental effort during incubation and nestling provisioning, each with 1h and 3h sampling windows were compiled from Solomon coding sheets using an Excel macro.

For each sex separately, we calculated the proportion of observation time when the following behaviours occurred (referred to as ‘parental effort’ henceforth): incubation (or brooding) and attending the nest; the latter was defined as the sum of time spent incubating (or brooding) and time spent inside the nest. In addition to individual effort, we also calculated the co-occurrence of these behaviours in the two sexes (‘joint incubation’, ‘joint brooding’ and ‘joint nest attendance’ henceforth). Total incubation, brooding and nest attendance were calculated as the time when at least one parent incubated, brooded, and attended the nest, respectively. Finally, proportion of male feedings were calculated as the ratio of the number of feedings by the male to the sum of feedings by the male and female, to control for between-nest differences in number of feeding visits arising from differences in brood size (cf. [11]). Zebra finches feed their nestlings by regurgitating food a number of times during each visit to the nest, and we quantified these from the recordings taken from the inside of the nest box.

Video recordings were coded by eight observers, therefore, behaviours were clearly and carefully defined so that different observers could unambiguously record the same behaviours accurately. Video recordings taken on day 8 of incubation of four out of ten nests were re-coded by a second observer to estimate inter-observer reliability. Parental effort (time spent incubating and attending the nest by the male, the female and both parents) coded by the two observers were correlated for each nest separately, to account for non-independence in the dataset. Correlation coefficients were then averaged over the four nests. Our results confirmed high correspondence between observers (mean ± SE of Pearson correlation coefficients, *r* = 0.91 ± 0.18; range: 0.64–0.99).

### Statistical Analyses

We analysed parental effort during incubation and nestling provisioning using the R statistical environment (v. 3.2.3; [34]). Division of labour was analysed in four separate Linear Mixed-effects Models (LMM; R package ‘lme4’ [35]), with proportion of time spent incubating (or brooding) and attending the nest (response variables) during incubation and nestling provisioning. The models included time in the reproductive stage (factor with three levels: early, middle and late) and parental sex (factor with two levels: male or female) as fixed factors, and cage ID and parent ID as nested random terms. We also tested for a two-way interaction between parental sex and time in reproductive stage because this would indicate changes in division of labour with advance of reproductive stage. Joint incubation and nest attendance, and total incubation and nest attendance were also analysed in separate LMMs, with time in reproductive stage as fixed factor and cage ID as random term. The effects of explanatory variables in all models were analysed by likelihood ratio tests.

Diurnal variation in parental effort was analysed by focusing on full-day recordings (on day 3 and 13 of incubation and day 3 and 17 post-hatching). First we analysed parental effort in separate LMMs with 1h sampling windows as periods. Initial investigation of the effect of period suggested a non-linear relationship, therefore we used AIC-based model selection to find the most suitable degree for the polynomial (tested range: 1–6). Besides the polynomial of period (covariate), the models included cage as a random term. Second, consistency of parental effort over the day was analysed by testing for repeatability [36, 37]. If the one-way ANOVA showed significantly larger between-individuals than within-individuals variance (i.e. our measurement was significantly repeatable), we continued with calculating the repeatability estimate following Harper [36]. Consistency of parental effort was analysed using 1h and 3h sampling windows for each day separately. In addition, for day 3 of incubation and nestling provisioning, we used linear regression to investigate how parental effort in a given period (1h or 3h) predicted overall daily parental effort. We compared *R*^2^ values from the linear models and obtained 95% confidence intervals for these estimates using non-parametric bootstrapping, in which we used random sampling with replacement and 10^4^ replicates (R-package ‘boot’; [38, 39]).

Reproductive stage-dependent within-individual variation of parental effort was analysed in two ways. Firstly, we focused on individual variation in parental effort within a given reproductive stage, during incubation and nestling provisioning, separately. Repeatability estimates were calculated using the 3h recordings (10:00–13:00) collected early, middle and late in each reproductive stage. Secondly, we analysed individual variation in parental effort between-reproductive stages by testing for repeatability of parental effort between day 8 of incubation and day 10 post-hatching. In this analysis, we compared corresponding parental efforts of the same parents collected during incubation (day 8, 10:00–13:00) with that collected during nestling provisioning (day 10 post-hatching, 10:00–13:00), for instance, incubation (incubation) compared with brooding (nestling provisioning).

### Ethical Note

Zebra finches used in the experiments remained for their entire life at the Department of Ethology, Eötvös Loránd University. Offspring produced during the experiment were recruited to our stock population. The study was carried out according to the Hungarian Laws for the experimentation with animals. Breeding and experimentation was carried out with permission from the Ethical Board of Eötvös Loránd University (# ELTE MÁB 02/2014).

## Results

### Parental Sex Roles in Zebra Finches

#### Division of Labour during Incubation

**Fig 2:**
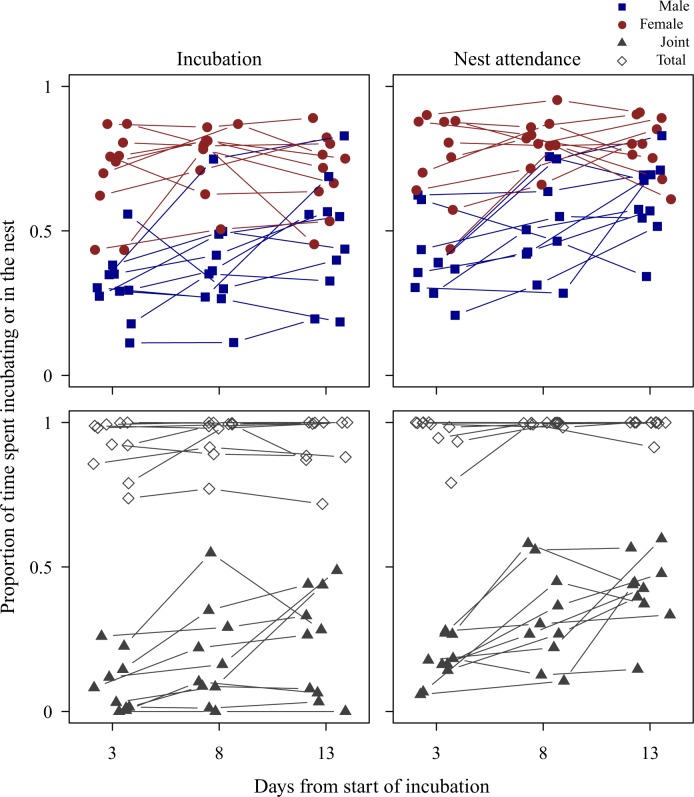
Division of labour and individual variation of parental effort in zebra finches during incubation. The figure shows proportion of 3h observation periods (10:00–13:00) spent incubating and inside the nest (nest attendance) during early, middle and late incubation (day 3, 8 and 13 of incubation, respectively). Blue filled squares represent male, red filled circles female, grey filled triangles joint (male and female simultaneous) and grey open diamonds total parental effort (when at least one parent incubates or attends the nest). Data from the same individuals are connected with lines.

Female zebra finches allocated more of their time to incubation and nest attendance than males (mean ± SE of male vs. female proportion of time spent incubating and attending the nest on day 3, 8 and 13 of incubation, combined: incubation: 0.39 ± 0.04 vs. 0.72 ± 0.03, *t*_18_ = -6.12, *P* < 0.001; nest attendance: 0.51 ± 0.03 vs. 0.78 ± 0.03, *t*_18_ = -6.55, *P* < 0.001; Fig 2). Inequality in division of labour was more pronounced during early than late incubation because male effort increased with advancing reproductive stage (LMM of incubation, parental sex x time in reproductive stage interaction: *χ*^2^_2_ = 6.00, *P* = 0.050; LMM of nest attendance, parental sex x time in reproductive stage interaction: *χ*^2^_2_ = 6.10, *P* = 0.047; Fig 2). Increasing male effort also resulted in an increase in joint incubation and nest attendance with advancing reproductive stage (LMM of joint incubation, time in reproductive stage: *χ*^2^_2_ = 9.57, *P* = 0.008; LMM of joint nest attendance, time in reproductive stage: *χ*^2^_2_ = 17.99, *P* < 0.001; Fig 2), although total incubation and nest attendance did not change (both *P* > 0.168).

#### Division of Labour during Nestling Provisioning

**Fig 3:**
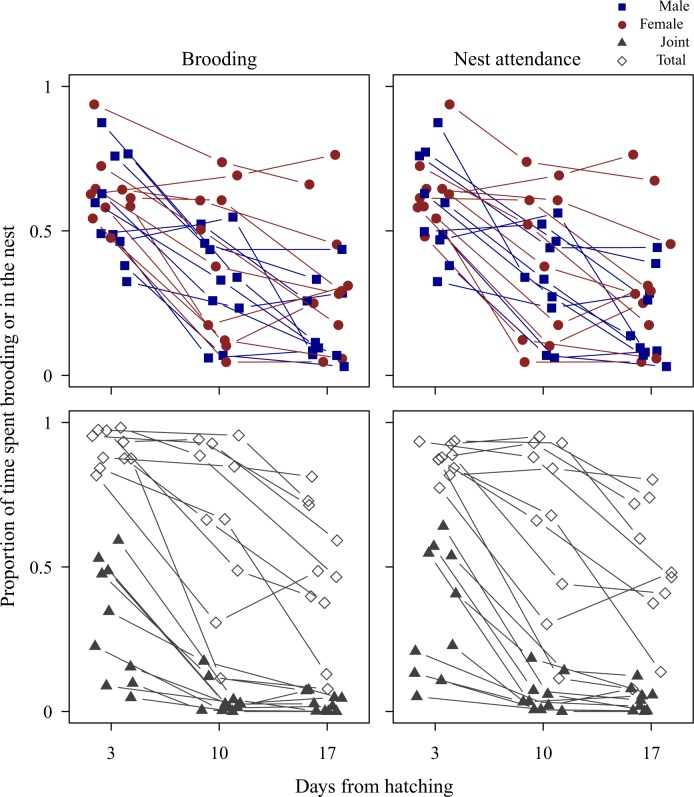
Division of labour and individual variation of parental effort in zebra finches during nestling provisioning. The figure shows proportion of 3h observation periods (10:00–13:00) spent brooding and inside the nest (nest attendance) during early, middle and late nestling provisioning (day 3, 10 and 17 post-hatching, respectively). Blue filled squares represent male, red filled circles female, grey filled triangles joint (male and female simultaneous) and grey open diamonds total parental effort (when at least one parent broods or attends the nest). Data from the same individuals are connected with lines.

Parental effort was shared more evenly between the sexes during nestling provisioning than during incubation (mean ± SE of total male vs. total female proportion of time spent brooding on day 3, 10 and 17 of nestling provisioning, combined: brooding: 0.37 ± 0.04 vs. 0.45 ± 0.06, *t*_18_ = -1.32, *P* = 0.229; Fig 3). Time spent brooding in males and females was not different (LMM of brooding, parental sex: *χ*^2^_1_ = 2.14, *P* = 0.144), but decreased with time in the reproductive stage in both sexes (time in reproductive stage: *χ*^2^_2_ = 44.52, *P* < 0.001). The decrease with reproductive stage was comparable in male and female parental effort, so that division of care did not change in interaction with advancing reproductive stage (all *P* > 0.144; Fig 3). Joint and total brooding also decreased with time in reproductive stage (LMM of joint brooding, time in reproductive stage: *χ*^2^_2_ = 23.31, *P* < 0.001; LMM of total brooding, time in reproductive stage: *χ*^2^_2_ = 21.11, *P* < 0.001; Fig 3).

The analysis of nest attendance revealed that brooding and nest attendance were highly and positively correlated during nestling provisioning (mean ± SE of Pearson’s correlation coefficients, brooding vs. nest attendance for male, female, joint and total parental effort on day 3 post-hatching: 0.97 ± 0.03; on day 17 post-hatching: 0.99 ± 0.01; Fig 3), so that in all further analyses we focused only on brooding and analyses were not repeated for nest attendance during nestling provisioning.

Males and females did not differ in how frequently they fed their young (mean ± SE proportion of male feedings (calculated as the number of times the male fed the young out of total feedings by the male and the female): 0.51 ± 0.04; one-sample *t*-test with true value of mean set to 0.5: *t*_29_ = 0.37, *P* = 0.712). Proportion of male feedings did not change with time in the reproductive stage (LMM, time in reproductive stage: *χ*^2^_2_ = 0.96, *P* = 0.618).

### Diurnal Variation in Parental Effort

#### Diurnal Variation in Parental Effort during Incubation

**Fig 4:**
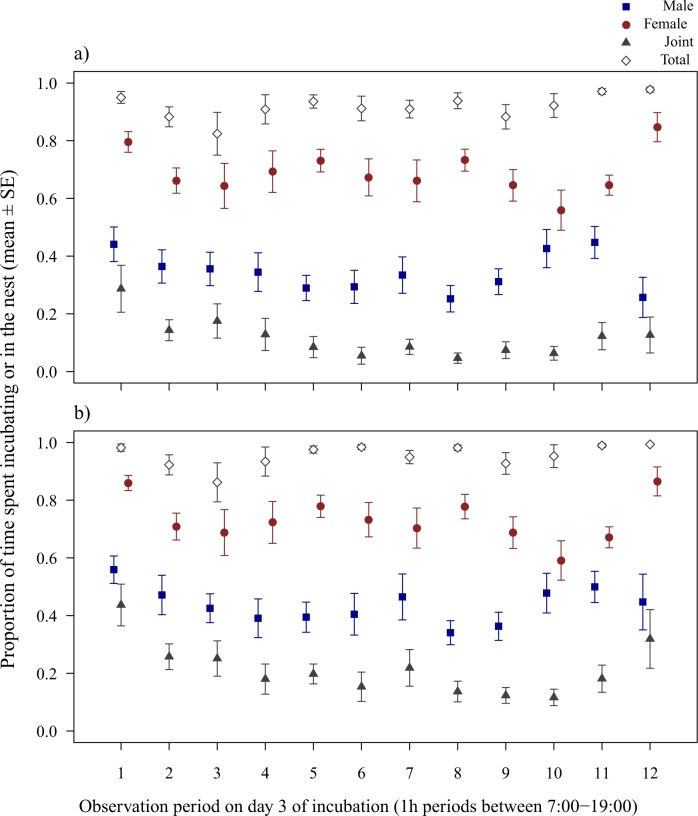
Diurnal variation of parental effort in zebra finches on day 3 of incubation. Mean ± SE proportion of 1h observation periods spent incubating (a) and in the nest (b) between 7:00–19:00. Blue filled squares represent male, red filled circles female, grey filled triangles joint (male and female simultaneous) and grey open diamonds total parental effort (when at least one parent incubates or attends the nest).

Male, female and joint incubation changed nonlinearly with daytime during early incubation (LMM of male incubation on day 3, 5^th^ degree polynomial of period: *χ*^2^_5_ = 16.84, *P* = 0.004; LMM of female incubation, 4^th^ degree polynomial of period: *χ*^2^_4_ = 19.87, *P* < 0.001; LMM of joint incubation, 2^nd^ degree polynomial of period: *χ*^2^_2_ = 31.60, *P* < 0.001; Fig 4A), whereas total incubation tended to increase linearly with period (LMM, *χ*^2^_1_ = 3.99, *P* = 0.046). Nest attendance on day 3 of incubation showed qualitatively similar patterns to incubation. Male, female and joint nest attendance changed nonlinearly with daytime (LMM of male attendance, 2^nd^ degree polynomial of period: *χ*^2^_2_ = 6.89, *P* = 0.032; LMM of female attendance, 4^th^ degree polynomial of period: *χ*^2^_4_ = 21.61, *P* < 0.001; LMM of joint attendance, 4^th^ degree polynomial of period: *χ*^2^_4_ = 29.81, *P* < 0.001; Fig 4B), whereas total attendance did not change with period (LMM, *χ*^2^_1_ = 2.92, *P* = 0.088).

During late incubation, however, we found no effect of period on incubation (male, female, joint or total incubation) or on nest attendance (with the exception of total nest attendance that increased with period; S1 Fig).

Despite the significant variation in parental effort over the day during early incubation (day 3), our repeatability analysis showed consistent behaviour for all parental efforts that were investigated, except for joint and total nest attendance (Table 1). For early incubation, repeatability estimates using 3h sampling windows were higher than estimates using 1h sampling windows (mean ± SE of repeatability estimates over 6 out of 8 parental efforts, 1h vs. 3h sampling windows, day 3 of incubation: 0.22 ± 0.03 vs. 0.40 ± 0.03, paired t-test: *t*_5_ = -11.93, *P* < 0.001; i.e. low vs. moderate repeatability based on [40]; Table 1). During late incubation (day 13), all parental efforts were consistent, including joint and total nest attendance. 3h sampling windows resulted in higher repeatability estimates for all parental efforts than 1h sampling windows (mean ± SE of repeatability estimates, 1h vs. 3h sampling windows, day 13 of incubation: 0.37 ± 0.08 vs. 0.57 ± 0.07, paired t-test: *t*_7_ = -7.93, *P* < 0.001, i.e. moderate repeatability in both cases; Table 1).

**Table 1:** Consistency of parental effort over the course of the day using 1h and 3h sampling windows on day 3 and day 13 of incubation, separately.

	1h sampling windows			3h sampling windows		
**D3 of incubation**	***r***	***F***_**9,110**_	***P***	***r***	***F***_**9,30**_	***P***
Male incubation	0.23	4.68	< 0.001	0.45	4.29	0.001
Male attendance	0.19	3.83	< 0.001	0.38	3.44	0.005
Female incubation	0.18	3.64	< 0.001	0.32	2.93	0.013
Female attendance	0.18	3.68	< 0.001	0.34	3.08	0.009
Joint incubation	0.36	7.84	< 0.001	0.49	4.78	< 0.001
Joint attendance		1.79	0.078		1.45	0.211
Total incubation	0.19	3.86	< 0.001	0.39	3.55	0.004
Total attendance		1.33	0.231		1.25	0.305
**D13 of incubation**	***r***	***F***_**9,107**_	***P***	***r***	***F***_**9,29**_	***P***
Male incubation	0.47	11.85	< 0.001	0.75	13.06	< 0.001
Male attendance	0.15	3.07	0.002	0.34	3.02	0.011
Female incubation	0.27	5.44	< 0.001	0.57	6.27	< 0.001
Female attendance	0.16	3.22	0.001	0.43	4.00	0.002
Joint incubation	0.58	17.44	< 0.001	0.75	12.81	< 0.001
Joint attendance	0.13	2.80	0.005	0.28	2.57	0.026
Total incubation	0.73	33.14	< 0.001	0.85	24.27	< 0.001
Total attendance	0.43	10.00	< 0.001	0.56	6.00	< 0.001

Repeatability estimates (r) were calculated only for parental efforts that were significantly repeatable.

Using 1h and 3h sampling windows, we investigated how parental effort at a given time of day predicts overall daily parental effort. Our analysis revealed that a single 1h sampling was not adequate for obtaining an accurate prediction for all parental efforts: daily male, female and total incubation were best predicted by a late afternoon, morning and midday sampling, respectively (Table 2). Joint incubation was the only exception since all 1h periods provided a good prediction of the overall daily joint incubation. In contrast with 1h periods, using 3h sampling windows provided good estimates for all parental efforts, independent of daytime (except for female and total incubation during late afternoon/evening i.e. in the last 3h period; Table 2). Apart from early morning samples for females, nest attendance with 1h periods showed no clear trends, only a few periods with good predictions scattered over the day among various parental efforts (Table 2). 3h periods again provided overall better estimates, with the exceptions of early morning sampling of male and late afternoon sampling of female attendance. Predictions of joint and total attendance were in contrast with each other: all but the early afternoon sample provided good estimates for joint attendance, however this was the only period that predicted total nest attendance (Table 2).

**Table 2:** Overall daily incubation and nest attendance of ten zebra finch pairs on day 3 of incubation predicted by a series of 1h and 3h sampling periods.

**Incubation**	**Male**		**Female**		**Joint**		**Total**	
**1h periods**	***R***^**2**^ **[95% CI]**	***P***	***R***^**2**^ **[95% CI]**	***P***	***R***^**2**^ **[95% CI]**	***P***	***R***^**2**^ **[95% CI]**	***P***
7:00–8:00	0.25 [0.00; 0.53]	0.138	0.15 [0.00; 0.67]	0.270	**0.52 [0.03; 0.81]**	**0.018**	**0.54 [0.06; 0.88]**	**0.016**
8:00–9:00	0.37 [0.00; 0.89]	0.060	**0.50 [0.01; 0.88]**	**0.023**	**0.66 [0.19; 0.92]**	**0.004**	0.28 [0.00; 0.65]	0.114
9:00–10:00	**0.57 [0.15; 0.78]**	**0.011**	**0.41 [0.03; 0.93]**	**0.044**	**0.88 [0.08; 0.95]**	**< 0.001**	0.23 [0.00; 0.90]	0.161
10:00–11:00	0.11 [0.00; 0.63]	0.343	0.25 [0.00; 0.58]	0.140	**0.42 [0.04; 0.87]**	**0.041**	0.16 [0.00; 0.85]	0.255
11:00–12:00	0.24 [0.00; 0.79]	0.147	0.29 [0.00; 0.71]	0.110	**0.51 [0.04; 0.87]**	**0.020**	**0.70 [0.28; 0.95]**	**0.002**
12:00–13:00	0.33 [0.00; 0.88]	0.081	**0.83 [0.19; 0.96]**	**< 0.001**	**0.49 [0.04; 0.68]**	**0.024**	**0.65 [0.07; 0.95]**	**0.005**
13:00–14:00	0.00 [0.00; 0.00]	0.957	0.33 [0.00; 0.83]	0.080	**0.46 [0.05; 0.97]**	**0.032**	**0.59 [0.17; 0.96]**	**0.009**
14:00–15:00	**0.84 [0.41; 0.91]**	**< 0.001**	0.27 [0.00; 0.86]	0.127	**0.54 [0.02; 0.85]**	**0.015**	**0.72 [0.11; 0.96]**	**0.002**
15:00–16:00	0.29 [0.00; 0.78]	0.109	**0.48 [0.00; 0.73]**	**0.027**	**0.47 [0.01; 0.83]**	**0.028**	0.39 [0.00; 0.80]	0.055
16:00–17:00	**0.41 [0.00; 0.91]**	**0.046**	0.28 [0.00; 0.77]	0.118	**0.75 [0.33; 0.96]**	**0.001**	0.04 [0.00; 0.88]	0.582
17:00–18:00	**0.59 [0.20; 0.88]**	**0.010**	0.02 [0.00; 0.25]	0.695	**0.75 [0.16; 0.93]**	**0.001**	0.19 [0.00; 0.78]	0.205
18:00–19:00	**0.61 [0.06; 0.78]**	**0.007**	0.06 [0.00; 0.46]	0.490	**0.42 [0.08; 0.73]**	**0.044**	**0.85 [0.35; 0.98]**	**0.000**
**3h periods**								
7:00–10:00	**0.72 [0.23; 0.90]**	**0.002**	**0.52 [0.05; 0.92]**	**0.019**	**0.80 [0.20; 0.96]**	**< 0.001**	**0.56 [0.01; 0.89]**	**0.013**
10:00–13:00	**0.47 [0.00; 0.90]**	**0.030**	**0.60 [0.12; 0.96]**	**0.009**	**0.84 [0.48; 0.95]**	**< 0.001**	**0.68 [0.32; 0.96]**	**0.003**
13:00–16:00	**0.44 [0.01; 0.87]**	**0.036**	**0.60 [0.01; 0.93]**	**0.009**	**0.65 [0.10; 0.86]**	**0.005**	**0.88 [0.37; 0.98]**	**< 0.001**
16:00–19:00	**0.85 [0.48; 0.97]**	**0.000**	0.19 [0.00; 0.65]	0.208	**0.82 [0.53; 0.94]**	**< 0.001**	0.22 [0.00; 0.90]	0.171
**Attendance**	**Male**		**Female**		**Joint**		**Total**	
**1h periods**	***R***^**2**^ **[95% CI]**	***P***	***R***^**2**^ **[95% CI]**	***P***	***R***^**2**^ **[95% CI]**	***P***	***R***^**2**^ **[95% CI]**	***P***
7:00–8:00	0.03 [0.00; 0.31]	0.609	**0.40 [0.02; 0.93]**	**0.050**	0.34 [0.00; 0.87]	0.077	0.39 [0.01; 0.85]	0.053
8:00–9:00	0.35 [0.00; 0.91]	0.072	**0.52 [0.00; 0.88]**	**0.018**	0.25 [0.00; 0.62]	0.146	**0.49 [0.04; 0.87]**	**0.024**
9:00–10:00	0.01 [0.00; 0.08]	0.818	0.38 [0.01; 0.73]	0.059	0.28 [0.00; 0.70]	0.114	0.04 [0.00; 0.36]	0.557
10:00–11:00	0.13 [0.00; 0.52]	0.297	0.30 [0.00; 0.53]	0.101	0.03 [0.00; 0.24]	0.651	0.36 [0.00; 0.74]	0.067
11:00–12:00	0.25 [0.00; 0.64]	0.143	0.30 [0.00; 0.57]	0.098	0.17 [0.00; 0.55]	0.241	0.28 [0.00; 0.72]	0.113
12:00–13:00	**0.46 [0.01; 0.84]**	**0.031**	**0.75 [0.35; 0.95]**	**0.001**	0.25 [0.00; 0.60]	0.141	0.00 [0.00; 0.03]	0.885
13:00–14:00	0.38 [0.01; 0.74]	0.057	0.07 [0.00; 0.50]	0.450	0.00 [0.00; 0.04]	0.873	**0.45 [0.00; 0.72]**	**0.034**
14:00–15:00	**0.74 [0.02; 0.95]**	**0.001**	0.39 [0.00; 0.81]	0.053	**0.46 [0.01; 0.73]**	**0.030**	0.09 [0.00; 0.63]	0.396
15:00–16:00	0.27 [0.01; 0.60]	0.125	0.39 [0.03; 0.81]	0.053	0.01 [0.00; 0.13]	0.761	**0.44 [0.03; 0.82]**	**0.036**
16:00–17:00	0.32 [0.00; 0.91]	0.086	0.35 [0.00; 0.96]	0.069	0.09 [0.00; 0.52]	0.395	0.00 [0.00; 0.01]	0.944
17:00–18:00	0.13 [0.00; 0.72]	0.309	0.01 [0.00; 0.14]	0.755	**0.64 [0.01; 0.86]**	**0.005**	0.02 [0.00; 0.25]	0.688
18:00–19:00	**0.68 [0.07; 0.86]**	**0.003**	0.12 [0.00; 0.48]	0.323	0.36 [0.00; 0.72]	0.069	**0.69 [0.11; 0.84]**	**0.003**
**3h periods**								
7:00–10:00	0.32 [0.02; 0.72]	0.090	**0.56 [0.06; 0.89]**	**0.013**	**0.60 [0.06; 0.93]**	**0.008**	0.37 [0.00; 0.81]	0.062
10:00–13:00	**0.63 [0.11; 0.84]**	**0.006**	**0.59 [0.20; 0.78]**	**0.010**	**0.42 [0.01; 0.90]**	**0.043**	0.34 [0.00; 0.64]	0.076
13:00–16:00	**0.66 [0.19; 0.85]**	**0.004**	**0.48 [0.03; 0.86]**	**0.027**	0.05 [0.00; 0.39]	0.524	**0.79 [0.19; 0.97]**	**< 0.001**
16:00–19:00	**0.69 [0.03; 0.95]**	**0.003**	0.34 [0.00; 0.88]	0.078	**0.63 [0.01; 0.80]**	**0.006**	0.01 [0.00; 0.12]	0.841

Proportion of variance explained (*R*^2^) with 95% confidence interval estimated from bootstrapping and statistical significance of the relationship between parental effort in the given period and during the full day are given. Significant relationships are highlighted in bold.

#### Diurnal Variation in Parental Effort during Nestling Provisioning

**Fig 5:**
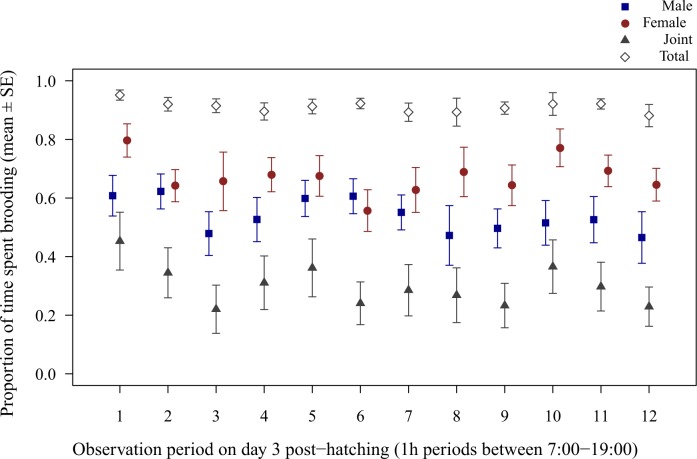
Diurnal variation of parental effort in zebra finches on day 3 post-hatching. Mean ± SE proportion of 1h observation periods spent brooding between 7:00–19:00. Blue filled squares represent male, red filled circles female, grey filled triangles joint (male and female simultaneous) and grey open diamonds total brooding (when at least one parent broods).

Male brooding decreased with increasing time of day during early nestling provisioning (LMM of male brooding on day 3 post-hatching, period: *χ*^2^_1_ = 5.45, *P* = 0.020) and there was a non-significant trend towards joint brooding decreasing with time of day (LMM of joint brooding, period: *χ*^2^_1_ = 3.80, *P* = 0.051; Fig 5). Female and total brooding, however, did not change significantly with time of day (both *P* > 0.161). During late nestling provisioning, no parental effort (including male, female, joint or total brooding and proportion of male feedings) changed with time of day (S2 Fig).

Repeatability analysis of parental effort on day 3 and day 17 post-hatching revealed consistent parental efforts within these days: all investigated parental efforts were consistent (Table 3). Similarly to incubation, repeatability estimates from 3h sampling windows were higher than estimates from 1h sampling windows for day 3 post-hatching (mean ± SE of repeatability estimates over 5 parental efforts, 1h vs. 3h sampling windows: 0.46 ± 0.06 vs. 0.70 ± 0.05, paired t-test: *t*_4_ = -9.92, *P* < 0.001, i.e. moderate vs. high repeatability; Table 3). For day 17 post-hatching, 3h periods also provided higher estimates for each parental effort than for 1h periods (mean ± SE of repeatability estimates, 1h vs. 3h sampling windows: 0.43 ± 0.05 vs. 0.54 ± 0.07, paired t-test: *t*_4_ = -4.49, *P* = 0.011, i.e. moderate repeatability in both cases; Table 3).

**Table 3:** Consistency of parental effort over the course of the day using 1h and 3h sampling windows on day 3 and day 17 post-hatching, separately.

	1h sampling windows			3h sampling windows		
**D3 post-hatching**	***r***	***F***_**9,110**_	***P***	***r***	***F***_**9,30**_	***P***
Male brooding	0.54	15.18	< 0.001	0.78	15.57	< 0.001
Female brooding	0.27	5.50	< 0.001	0.58	6.46	< 0.001
Joint brooding	0.62	20.47	< 0.001	0.86	24.95	< 0.001
Total brooding	0.39	8.67	< 0.001	0.62	7.50	< 0.001
Proportion of male feedings	0.48	5.39	< 0.001	0.64	8.14	< 0.001
**D17 post-hatching**	***r***	***F***_**9,110**_	***P***	***r***	***F***_**9,30**_	***P***
Male brooding	0.48	12.03	< 0.001	0.57	6.23	< 0.001
Female brooding	0.54	15.26	< 0.001	0.69	9.77	< 0.001
Joint brooding	0.25	4.91	< 0.001	0.28	2.57	0.025
Total brooding	0.49	12.72	< 0.001	0.60	7.05	< 0.001
Proportion of male feedings	0.40	6.43	< 0.001	0.57	6.34	< 0.001

Repeatability estimates (*r*) are given and results of the one-way ANOVA.

Our analysis of how parental effort, sampled using 1h and 3h sampling windows at a given time of day predicted overall daily parental effort showed that overall daily male and joint brooding was significantly predicted by 1h samples taken at various times of day for all but two periods for both variables (Table 4). For female and total brooding, and proportion of male feedings, 6, 8 and 5 out of 12 periods, respectively, predicted overall daily effort. Periods explaining the largest proportion of variance appeared to be scattered randomly over the day. Similarly to all previous analyses, parental efforts sampled in 3h periods resulted in a larger proportion of the variance in overall daily effort being explained, and this was independent of time of day (Table 4).

**Table 4:** Overall daily brooding and proportion of male feedings in ten zebra finch pairs on day 3 post-hatching predicted by 1h and 3h sampling periods.

Brooding	Male		Female		Joint		Total		Proportion of male feedings	
**1h periods**	***R***^**2**^ **[95% CI]**	***P***	***R***^**2**^ **[95% CI]**	***P***	***R***^**2**^ **[95% CI]**	***P***	***R***^**2**^ **[95% CI]**	***P***	***R***^**2**^ **[95% CI]**	***P***
7:00–8:00	**0.71 [0.07; 0.95]**	**0.002**	**0.50 [0.01; 0.81]**	**0.021**	**0.88 [0.54; 0.96]**	**< 0.001**	**0.43 [0.01; 0.90]**	**0.038**	0.44 [0.05; 0.96]	0.051
8:00–9:00	**0.64 [0.22; 0.85]**	**0.005**	**0.48 [0.03; 0.77]**	**0.027**	**0.74 [0.26; 0.93]**	**0.001**	**0.71 [0.14; 0.91]**	**0.002**	0.10 [0.00; 0.87]	0.375
9:00–10:00	0.18 [0.00; 0.63]	0.221	0.39 [0.00; 0.82]	0.053	**0.55 [0.02; 0.89]**	**0.015**	0.00 [0.00; 0.04]	0.850	0.36 [0.02; 0.68]	0.066
10:00–11:00	**0.63 [0.23; 0.87]**	**0.006**	**0.68 [0.26; 0.87]**	**0.003**	**0.62 [0.09; 0.92]**	**0.007**	**0.57 [0.14; 0.84]**	**0.011**	**0.84 [0.37; 0.92]**	**< 0.001**
11:00–12:00	**0.73 [0.27; 0.91]**	**0.002**	0.31 [0.00; 0.74]	0.092	**0.64 [0.05; 0.88]**	**0.005**	**0.41 [0.01; 0.89]**	**0.048**	**0.85 [0.06; 0.93]**	**< 0.001**
12:00–13:00	**0.70 [0.31; 0.89]**	**0.003**	0.02 [0.00; 0.18]	0.672	0.16 [0.00; 0.82]	0.250	0.33 [0.02; 0.72]	0.083	0.17 [0.00; 0.71]	0.238
13:00–14:00	0.38 [0.01; 0.78]	0.059	**0.54 [0.14; 0.85]**	**0.015**	**0.77 [0.15; 0.93]**	**< 0.001**	**0.56 [0.02; 0.89]**	**0.013**	**0.85 [0.56; 0.98]**	**< 0.001**
14:00–15:00	**0.61 [0.19; 0.88]**	**0.007**	**0.71 [0.06; 0.97]**	**0.002**	**0.91 [0.67; 0.96]**	**< 0.001**	**0.91 [0.77; 0.97]**	**< 0.001**	0.00 [0.00; 0.03]	0.885
15:00–16:00	**0.64 [0.11; 0.91]**	**0.006**	0.25 [0.00; 0.76]	0.144	**0.82 [0.47; 0.91]**	**< 0.001**	0.03 [0.00; 0.34]	0.658	**0.46 [0.04; 0.76]**	**0.032**
16:00–17:00	**0.79 [0.18; 0.96]**	**0.001**	**0.75 [0.29; 0.88]**	**0.001**	**0.89 [0.60; 0.99]**	**< 0.001**	**0.72 [0.23; 0.91]**	**0.002**	0.28 [0.01; 0.73]	0.117
17:00–18:00	**0.78 [0.19; 0.92]**	**0.001**	0.00 [0.00; 0.00]	0.933	0.46 [0.01; 0.90]	0.032	0.28 [0.00; 0.61]	0.115	**0.60 [0.01; 0.91]**	**0.014**
18:00–19:00	**0.65 [0.06; 0.93]**	**0.005**	0.18 [0.00; 0.82]	0.224	**0.84 [0.62; 0.95]**	**< 0.001**	**0.80 [0.19; 0.95]**	**< 0.001**	0.12 [0.00; 0.63]	0.332
**3h periods**										
7:00–10:00	**0.90 [0.49; 0.97]**	**< 0.001**	**0.76 [0.39; 0.93]**	**0.001**	**0.95 [0.78; 0.99]**	**< 0.001**	**0.49 [0.02; 0.77]**	**0.024**	**0.68 [0.01; 0.86]**	**0.003**
10:00–13:00	**0.90 [0.70; 0.96]**	**< 0.001**	**0.69 [0.10; 0.86]**	**0.003**	**0.85 [0.58; 0.94]**	**< 0.001**	**0.73 [0.23; 0.92]**	**0.002**	**0.93 [0.68; 0.98]**	**< 0.001**
13:00–16:00	**0.81 [0.19; 0.94]**	**< 0.001**	**0.76 [0.29; 0.92]**	**0.001**	**0.95 [0.83; 0.99]**	**< 0.001**	**0.86 [0.28; 0.94]**	**< 0.001**	**0.73 [0.31; 0.90]**	**0.002**
16:00–19:00	**0.89 [0.46; 0.98]**	**< 0.001**	**0.65 [0.26; 0.90]**	**0.005**	**0.84 [0.57; 0.99]**	**< 0.001**	**0.83 [0.49; 0.94]**	**< 0.001**	**0.65 [0.02; 0.94]**	**0.005**

Proportion of variance explained (*R*^2^) with 95% confidence interval estimated from bootstrapping and statistical significance of the relationship between parental effort in the given period and during the full day are given. Significant relationships are highlighted in bold.

### Reproductive Stage-Dependent Individual Variation in Parental Effort

#### Individual Variation in Parental Effort within Reproductive Stages

Although male (and total) incubation increased from early to middle to late incubation (see division of labour analyses and Fig 2), incubation efforts (including male, female, joint and total incubation) were significantly and moderately repeatable (Table 5; S3 Fig). Nest attendance, however, was more variable within individuals, with only female attendance being consistent over early, middle and late incubation (Table 5; S3 Fig).

**Table 5:** Consistency of parental effort within reproductive stages in zebra finch parents.

**Repeatability over incubation**	***r***	***F***_**9,20**_	***P***
Male incubation	0.35	2.64	0.034
Male attendance		1.18	0.361
Female incubation	0.40	3.03	0.019
Female attendance	0.34	2.52	0.041
Joint incubation	0.42	3.26	0.013
Joint attendance		1.03	0.449
Total incubation	0.71	8.48	< 0.001
Total attendance		0.87	0.564
**Repeatability over nestling provisioning**	***r***	***F***_**9,20**_	***P***
Male brooding		0.83	0.596
Female brooding	0.34	2.58	0.038
Joint brooding		0.52	0.842
Total brooding		1.31	0.293
Proportion of male feedings		1.93	0.106

Periods with a 3h sampling window between 10:00–13:00 on day 3, 8 and 13 of incubation and on day 3, 10 and 17 post-hatching were analysed separately. Repeatability estimates were calculated only for parental efforts that were significantly repeatable.

During nestling provisioning, only female brooding was consistent, whereas all other parental efforts showed large within-individual variation over the reproductive stage (Table 5; S4 Fig).

#### Individual Variation in Parental Effort between Reproductive Stages

Parental effort between reproductive stages (3h samples taken from the same nests on day 8 of incubation vs. day 10 post-hatching, each between 10:00–13:00) were not consistent (for male, female, joint and total incubation/brooding and nest attendance, all *P* > 0.633; S5 Fig).

## Discussion

Our study investigated between- and within-individual variation in various forms of parental effort in captive zebra finches. We report changing division of labour with advancing breeding stage from a female-biased parental effort during incubation to a balanced division during nestling provisioning. Our results provide a detailed analysis of how well our sample, using different sampling windows, predicted overall parental effort in light of variation in parental effort within the reproductive stage, a given day and between vs. within individuals.

Parental effort was not shared evenly between the sexes during incubation. Female zebra finches allocated more of their time to incubation and attending the nest than males. This is in line with previous studies reporting female-biased care during incubation in captive, but not in free-living populations [28, 41–43]. Our study, besides corroborating the bias in contrast with recently published similar parental efforts from captive males and females [44], further highlights that this difference is not consistent within the reproductive stage (see also [28]), as it was more pronounced during early incubation and decreased over the reproductive stage. The existence and change of female-biased incubation was likely explained by sex differences in effectiveness of heat transition due to morphology [43, 45]. In zebra finches, only females develop a brood patch [43] and this results in more efficient heat transfer in females than in males, as found in a recent experiment [45]. Therefore, a change in female-biased incubation may reflect different sensitivity of embryogenesis for thermal conditions with advancing reproductive stage [46]. In addition, asymmetries in nest attendance suggests that other parental efforts involved in this reproductive stage but not investigated here (e.g. nest construction) also likely to be asymmetric.

Change in division of labour over the reproductive stage (and consistency of parental effort) has important implications for studies focusing on how parental effort is shared. It may be useful to adjust timing of sampling or manipulation based on this pattern, depending on the research question. For instance, if the study aims at using a single-sample approach, parental behaviour should be sampled at the middle of incubation. By contrast, if manipulation of incubation effort in males (or females) is needed (such as in many studies focusing on sexual conflict over parental care [10, 47]), it may be more fruitful to focus on the days with the most or the least-expressed sex difference in parental effort (depending on which parent is manipulated).

After hatching of the nestlings, such biased division of labour was not apparent: males and females put similar effort into brooding and feeding nestlings. Brooding time decreased with advancing reproductive stage, most likely in relation with changing thermoregulatory needs of the young [48, 49]. Thermo-regulation progresses gradually with feather growth, nevertheless, most of nest attendance involved brooding even during late nestling provisioning; feedings occurred during brooding when the parents regurgitated food. In addition to brooding, we found no sex differences in how frequently parents fed their young, and such equal division of labour was maintained during the reproductive stage.

Partitioning full-day recordings of parental effort during early and late incubation and nestling provisioning revealed significant diurnal variation early in each reproductive stage. We suggest this again may be related to change in thermoregulatory needs of embryos and nestlings with advancing reproductive stages. Diurnal changes were more apparent during incubation than during nestling provisioning, suggesting that young embryos may be more sensitive to low temperatures or temperature fluctuations than recently hatched young. In addition male, but not female brooding varied with time of day possibly due to sex differences in capabilities to transfer heat (cf. [45]).

We found individually consistent differences for most parental behaviours within given days of incubation, and in all parental behaviours within days of nestling provisioning. Using 3h sampling period provided better estimates of parental effort, reflected in higher repeatability estimates, and we suggest this was due to shorter sampling window (1h) estimates being more susceptible to stochastic and diurnal variation. Our analysis of how well a sample collected in a given period predicted overall daily effort confirmed this: using 3h sampling windows resulted in a higher proportion of variance explained in various parental efforts for both incubation and nestling provisioning. Taken together, results from these two analyses suggest a longer (3h) sampling window should be preferred whenever a single sampling per breeding pair is planned. Our results suggest that time of sampling in the day has very little effect on estimating overall parental effort.

Within-individual variation over the reproductive stage, on the other hand, should be taken into account, depending on the parental effort in question. Incubation was consistent between early, middle and late incubation, but nest attendance (except for female attendance) varied over the reproductive stage. Parental effort during nestling provisioning showed even higher within-individual variation, with female brooding being the only parental effort that was consistent. We found, therefore, that 3h sampling starting at a standardized time of day did not provide an accurate estimate for overall parental effort during nestling provisioning. This finding has been corroborated by the between-reproductive stage consistency analysis: parental effort during incubation did not predict parental effort during nestling provisioning. We suggest longer sampling on multiple days (best achieved by an automated monitoring system, e.g. [14]) may be needed for estimating overall parental effort accurately during nestling provisioning.

## Conclusions

We found division of labour to change within and between reproductive stages and to vary within a given day in our captive zebra finch population. Based on our analyses of within-individual variation and how our sampling method affects prediction of overall parental effort, we suggest that using longer sampling windows (3h) may be worth the effort as they can provide more consistent and accurate estimates of overall parental effort, while eliminating the effects of diurnal variation. Our results suggest that a single sample during incubation may provide accurate estimates of overall parental effort, although change with the reproductive stage needs to be considered. Our study revealed large within-individual variation during nestling provisioning which makes accurate estimation of overall parental effort during this reproductive stage more challenging.

## Supporting Information

S1 Fig Diurnal variation of parental effort in zebra finches on day 13 of incubation. Mean ± SE proportion of 1h observation periods spent incubating (a) and in the nest (b) between 7:00–19:00. Blue filled squares represent male, red filled circles female, grey filled triangles joint (male and female simultaneous), and grey open diamonds total parental effort (when at least one parent incubates or attends the nest). Male, female, joint and total incubation changed less with daytime than during early incubation (separate LMMs for each response variable, period in all: *P* > 0.067). None of male, female and joint nest attendance changed with daytime (separate LMMs for each response variable, period in all: *P* > 0.156), whereas total attendance increased with period (LMM, *χ*^2^_1_ = 4.59, *P* = 0.032).
(EPS)

S2 Fig Diurnal variation of parental effort in zebra finches on day 17 post-hatching. Mean ± SE proportion of 1h observation periods spent brooding between 7:00–19:00. Blue filled squares represent male, red filled circles female, grey filled triangles joint (male and female simultaneous), and grey open diamonds total brooding (when at least one parent broods). None of the investigated parental efforts (including proportion of male feedings, not shown on figure) changed significantly with daytime during late nestling provisioning (separate LMMs for each response variable, period in all: *P* > 0.068).
(EPS)

S3 Fig Within-reproductive stage consistency of parental effort during incubation in zebra finches. The figure shows the proportion of 3h periods spent incubating and inside the nest (nest attendance) during corresponding periods of late vs. early incubation (day 13 vs. day 3 of incubation). Blue filled squares represent male, red filled circles female, grey filled triangles joint (male and female simultaneous), and grey open diamonds total parental effort (when at least one parent incubates or attends the nest).
(EPS)

S4 Fig Within-reproductive stage consistency of parental effort during nestling provisioning in zebra finches. The figure shows the proportion of 3h periods spent brooding and inside the nest (nest attendance) during corresponding periods of late vs. early nestling provisioning (day 17 vs. day 3 post-hatching). Blue filled squares represent male, red filled circles female, grey filled triangles joint (male and female simultaneous), and grey open diamonds total parental effort (when at least one parent broods or attends the nest).
(EPS)

S5 Fig Between-reproductive stage consistency of parental effort in zebra finches. The figure illustrates the proportion of corresponding 3h observation periods (10:00–13:00) spent incubating and brooding (left panes) and inside the nest (right panes) on day 10 post-hatching vs. day 8 of incubation.
(EPS)

S1 Table Behavioural data. (XLSX)

## References

[1] Clutton-Brock TH. The evolution of parental care Princeton: Princeton University Press; 1991.

[2] Reynolds JD, Goodwin NB, Freckleton RP. Evolutionary transitions in parental care and live bearing in vertebrates. Philos Trans R Soc Lond B. 2002;357: 269–281. 11958696 10.1098/rstb.2001.0930 PMC1692951

[3] Royle NJ, Smiseth PT, Kölliker M, editors. The evolution of parental care Oxford: Oxford University Press; 2012.

[4] Klug H, Bonsall MB. What are the benefits of parental care? The importance of parental effects on developmental rate. Ecol Evol. 2014;4: 2330–2351. 10.1002/ece3.1083 25360271 PMC4203283

[5] Parker GA, Royle NJ, Hartley IR. Intrafamilial conflict and parental investment: a synthesis. Philos Trans R Soc Lond B. 2002;357: 295–307. 11958698 10.1098/rstb.2001.0950 PMC1692944

[6] Cockburn A. Prevalence of different modes of parental care in birds. Proc R Soc Lond B. 2006;273: 1375–1383. 10.1098/rspb.2005.3458 PMC1560291 16777726

[7] Gilby AJ, Mainwaring MC, Rollins LA, Griffith SC. Parental care in wild and captive zebra finches: measuring food delivery to quantify parental effort. Anim Behav. 2011;81: 289–295.

[8] Kosztolányi A, Cuthill IC, Székely T. Negotiation between parents over care: reversible compensation during incubation. Behav Ecol. 2009;20: 446–452.

[9] Grunst ML, Rotenberry JT, Grunst AS. Elevating perceived predation risk modifies the relationship between parental effort and song complexity in the song sparrow *Melospiza melodia*. J Avian Biol. 2016;47: 57–68.

[10] Pogány Á, Szurovecz Z, Vincze E, Barta Z, Székely T. Mate preference does not influence reproductive motivation and parental cooperation in female zebra finches. Behaviour. 2014;151: 1885–1901.

[11] Pogány Á, van Dijk RE, Horváth P, Székely T. Parental behavior and reproductive output in male-only cared and female-only cared clutches in the Eurasian penduline tit (*Remiz pendulinus*). Auk. 2012;129: 773–781.

[12] Bartlett TL, Mock DW, Schwagmeyer PL. Division of labor: Incubation and biparental care in house sparrows (*Passer domesticus*). Auk. 2005;122: 835–842.

[13] Murphy MT, Chutter CM, Redmond LJ. Quantification of avian parental behavior: what are the minimum necessary sample times? J Field Ornithol. 2015;86: 41–50.

[14] Lendvai ÁZ, Akcay C, Ouyang JQ, Dakin R, Domalik AD, St John PS, et al Analysis of the optimal duration of behavioral observations based on an automated continuous monitoring system in tree swallows (*Tachycineta bicolor*): Is one hour good enough? Plos One. 2015;10: e0141194 10.1371/journal.pone.0141194 26559407 PMC4641651

[15] AlRashidi M, Kosztolányi A, Küpper C, Cuthill IC, Javed S, Székely T. The influence of a hot environment on parental cooperation of a ground-nesting shorebird, the Kentish plover *Charadrius alexandrinus*. Front Zool. 2010;7: 1 10.1186/1742-9994-7-1 20148101 PMC2819062

[16] Riebel K. Song and female mate choice in zebra finches—a review. Adv Study Behav. 2009;40: 197–238.

[17] Royle NJ, Hartley IR, Parker GA. Sexual conflict reduces offspring fitness in zebra finches. Nature. 2002;416: 733–736. 10.1038/416733a 11961554

[18] Zann RA. The zebra finch: a synthesis of field and laboratory studies Oxford: Oxford University Press; 1996.

[19] Griffith SC, Buchanan KL. The zebra finch: the ultimate Australian supermodel. Emu. 2010;110: v–xii.

[20] Gilby AJ, Mainwaring MC, Griffith SC. The adaptive benefit of hatching asynchrony in wild zebra finches. Anim Behav. 2011;82: 479–484.

[21] Mariette MM, Pariser EC, Gilby AJ, Magrath MJL, Pryke SR, Griffith SC. Using an electronic monitoring system to link offspring provisioning and foraging behavior of a wild passerine. Auk. 2011;128: 26–35.

[22] Pooley EL, Kennedy MW, Nager RG. Maternal inbreeding reduces parental care in the zebra finch, *Taeniopygia guttata*. Anim Behav. 2014;97: 153–163.

[23] Mariette MM, Griffith SC. The adaptive significance of provisioning and foraging coordination between breeding partners. Am Nat. 2015;185: 270–280. 10.1086/679441 25616144

[24] Foster VS, Burley NT. Sex allocation in response to maternal condition: different tactics of care-giving by male and female zebra finches. Ethology. 2007;113: 511–520.

[25] Mariette MM, Griffith SC. Nest visit synchrony is high and correlates with reproductive success in the wild zebra finch *Taeniopygia guttata*. J Avian Biol. 2012;43: 131–140.

[26] Rehling A, Spiller I, Krause ET, Nager RG, Monaghan P, Trillmich F. Flexibility in the duration of parental care: zebra finch parents respond to offspring needs. Anim Behav. 2012;83: 35–39.

[27] Royle NJ, Hartley IR, Parker GA. Consequences of biparental care for begging and growth in zebra finches, *Taeniopygia guttata*. Anim Behav. 2006;72: 123–130.

[28] Gorman HE, Nager RG. State-dependent incubation behaviour in the zebra finch. Anim Behav. 2003;65: 745–54.

[29] Gorman HE, Arnold KE, Nager RG. Incubation effort in relation to male attractiveness in zebra finches *Taeniopygia guttata*. J Avian Biol. 2005;36: 413–420.

[30] Mainwaring MC, Lucy D, Hartley IR. Parentally biased favouritism in relation to offspring sex in zebra finches. Behav Ecol Sociobiol. 2011;65: 2261–2268.

[31] Arnold KE, Gilbert L, Gorman HE, Griffiths KJ, Adam A, Nager RG. Paternal attractiveness and the effects of differential allocation of parental investment. Anim Behav. 2016;113: 69–78.

[32] Forstmeier W, Segelbacher G, Mueller JC, Kempenaers B. Genetic variation and differentiation in captive and wild zebra finches (*Taeniopygia guttata*). Mol Ecol. 2007;16: 4039–4050. 10.1111/j.1365-294X.2007.03444.x 17894758

[33] Péter A. Solomon Coder: a simple solution for behavior coding. v 15.11.19. 2015; URL: http://solomoncoder.com/

[34] R Core Team. R: A language and environment for statistical computing. v. 3.2.3 Vienna, Austria: R Foundation for Statistical Computing; 2015.

[35] Bates D, Maechler M, Bolker B, Walker S. Fitting linear mixed-effects models using lme4. J Stat Softw. 2015;67: 1–48. R package v. 1.3–17.

[36] Harper DGC. Some comments on the repeatability of measurements. Ring Migr. 1994;15: 84–90.

[37] Lessells CM, Boag PT. Unrepeatable repeatabilities—a common mistake. Auk. 1987;104: 116–21.

[38] Canty A, Ripley B. boot: Bootstrap R (S-Plus) functions. R package v. 1.3–17. 2015.

[39] Davison AC, Hinkley DV. Bootstrap methods and their applications Cambridge: Cambridge University Press; 1997.

[40] Martin P, Bateson P. Measuring behavior: an introductory guide Cambridge: Cambridge University Press; 1986.

[41] Burley NT. The differential-allocation hypothesis—an experimental test. Am Nat. 1988;132: 611–628.

[42] Hill DL, Lindstrom J, Nager RG. Carry-over effects of male extra-pair copulation opportunity on biparental effort in zebra finches. Behav Ecol Sociobiol. 2011;65: 2049–2059.

[43] Zann R, Rossetto M. Zebra finch incubation—brood patch, egg temperature and thermal properties of the nest. Emu. 1991;91: 107–120.

[44] Gilby AJ, Mainwaring MC, Griffith SC. Incubation behaviour and hatching synchrony differ in wild and captive populations of the zebra finch. Anim Behav. 2013;85: 1329–1334.

[45] Hill DL, Lindstrom J, McCafferty DJ, Nager RG. Female but not male zebra finches adjust heat output in response to increased incubation demand. J Exp Biol. 2014;217: 1326–1332. 10.1242/jeb.095323 24363422

[46] Suarez ME, Wilson HR, McPherson BN, Mather FB, Wilcox CJ. Low temperature effects on embryonic development and hatch time. Poult Sci. 1996;75: 924–932. 8966182 10.3382/ps.0750924

[47] Harrison F, Barta Z, Cuthill I, Székely T. How is sexual conflict over parental care resolved? A meta-analysis. J Evol Biol. 2009;22: 1800–1812. 10.1111/j.1420-9101.2009.01792.x 19583699

[48] Walter I, Seebacher F. Endothermy in birds: underlying molecular mechanisms. J Exp Biol. 2009;212: 2328–2336. 10.1242/jeb.029009 19617425

[49] Tzschentke B, Nichelmann M. Development of avian thermoregulatory system during the early postnatal period: development of the thermoregulatory set-point. Ornis Fenn. 1999;76: 189–198.

